# Conditional Reduction of Predation Risk Associated with a Facultative Symbiont in an Insect

**DOI:** 10.1371/journal.pone.0143728

**Published:** 2015-11-30

**Authors:** Sarah Polin, Jean-François Le Gallic, Jean-Christophe Simon, Tsutomu Tsuchida, Yannick Outreman

**Affiliations:** 1 UMR 1349 IGEPP, Agrocampus Ouest, 35042, Rennes, France; 2 UMR 1349 IGEPP, INRA, 35653, Le Rheu, France; 3 Frontier Research Core for Life Sciences, University of Toyama, Toyama, 930–8555, Japan; 4 Université Européenne de Bretagne, Rennes, France; CSIRO, AUSTRALIA

## Abstract

Symbionts are widespread among eukaryotes and their impacts on the ecology and evolution of their hosts are meaningful. Most insects harbour obligate and facultative symbiotic bacteria that can influence their phenotype. In the pea aphid *Acyrthosiphon pisum*, an astounding symbiotic-mediated phenotype has been recently observed: when infected with the symbiotic bacteria *Rickettsiella viridis*, young red aphid larvae become greener at adulthood and even darker green when co-infected with *Rickettsiella viridis* and *Hamiltonella defensa*. As body colour affects the susceptibility towards natural enemies in aphids, the influence of the colour change due to these facultative symbionts on the host survival in presence of predators was tested. Our results suggested that the *Rickettsiella viridis* infection may impact positively host survival by reducing predation risk. Due to results from uninfected aphids (i.e., more green ones attacked), the main assumption is that this symbiotic infection would deter the predatory ladybird feeding by reducing the profitability of their hosts rather than decreasing host detection through body colour change. Aphids co-infected with *Rickettsiella viridis* and *Hamiltonella defensa* were, however, more exposed to predation suggesting an ecological cost associated with multiple infections. The underlying mechanisms and ecological consequences of these symbiotic effects are discussed.

## Introduction

While considered until recently as a marginal phenomenon, symbiotic associations are gaining recognition as a ubiquitous feature of animal life [1]. These associations with symbiotic microorganisms are widespread among animals and those microbial associates are often heritable, transmitted with high fidelity from parent to offspring. Because host species and their heritable symbionts share fates, but not necessarily common interests, inherited symbionts often exert phenotypical effects that can profoundly influence the ecology and the evolution of animal hosts [2]. These symbiont-mediated phenotypes can alter, negatively or positively, the interaction between the symbiont host and its natural enemies (i.e., pathogens, parasite and predators) [3]. Symbiont infection can be then associated with an increased susceptibility to pathogens explained by a weaker host immune system [4]. Inversely, symbiotic microbes can provide their hosts with a higher resistance to pathogens (i.e. bacteria, fungi or viruses) [5]; the microbial production of secondary metabolites with known toxic functions is often taken as prima facie evidence of these protective symbioses [6]. For instance, antimicrobial activity of the host is provided by the bacterial symbiont in hoopoe preen [7]. Less frequently, the microbial symbioses confer an efficient protection against predation: symbiont-mediated protection against predators have been only observed in Bryozoa, whereby a symbiont confers a chemical defence by excreting bryostatin that protects *Bugula neritina* larvae against fish predation [8], in bobtail squid, whereby the bioluminescence due its symbiotic bacterium, *Vibrio fisheri* provides a defensive camouflage strategy [9] and in *Paederus* beetle, whereby its bacterial symbiont synthesizes a chemical toxin that the insect can use as a defence against predators [10]. Are predation protective symbioses common and widespread, or do they occur only in a few specific systems with particular organisms and habitats? This opened question is crucial because if symbiotic associations strongly influence the prey-predator interaction outcomes, this may strongly influence the ecological and evolutionary processes in ecosystems.

Many insects harbour various types of microbial symbionts and would therefore be good candidates to test symbiont-mediated protection against predators. In aphids, in addition to their obligate nutrient-providing symbiont *Buchnera aphidicola*, many species also carry one or a few facultative bacterial symbionts that are mainly maternally inherited [11]. The extended phenotypes associated with these symbioses cover a large range of features from feeding facilitation and host plant adaptation [12] to resistance to biotic (i.e. pathogens [13] and parasites [14]) and abiotic stresses (i.e., heat tolerance [15]). In the pea aphid, *Acyrthosiphon pisum*, an astounding extended phenotype has recently been reported: a body colour change is associated with the infection with the gamma-proteobacterium *Rickettsiella viridis* [16], [17]. *Rickettsiella* bacterial genus is commonly associated with large costs for its host and therefore classified as a virulent pathogen. For the *Acyrthosiphon pisum*/*Rickettsiella viridis* association, surprisingly no associated cost has been highlighted [17], except for short longevity in some aphid clones [16].

In the pea aphid, red and green clones are usually found within the same populations. The extended phenotype associated with *Rickettsiella viridis* is easily noticeable in red clones: the young red larvae become greener as they grow and reach adulthood as green individuals. In natural populations, *Rickettsiella viridis* is commonly found in association with *Hamiltonella defensa* [16], a gamma-proteobacterium conferring a resistance against insect parasitoids [3]. In case of co-infection, the aphid individuals are even greener than *Rickettsiella*-infected aphids [17]. In nature, the ecological selective pressures may vary according to the colour of aphid individuals. While insect parasitoids are more likely to parasitize green aphids [18], [19], the predatory ladybirds tend to prefer red ones [20], [21]. Since the discovery of the *Rickettsiella*-associated extended phenotype, it has been hypothesised that the colour change would be adaptive by reducing the predation risk by ladybirds [16]. To investigate this assumption, we present data from experiments designed to test whether pea aphids harbouring *Rickettsiella*, singly or with *Hamiltonella*, suffer a less predation risk in presence of foraging ladybirds. For this purpose, the predators were exposed to different types of pea aphids, differing by their colour or/and symbiotic complement.

## Material and Methods

### Biological materials

#### Aphids

Table 1 presents all pea aphid strains used for our study. Four different *A*. *pisum* aphid types were defined according to their colour and symbiotic complement: (*i*) red aphid genotypes free of facultative bacterial symbionts, (*ii*) green aphid genotypes free of facultative bacterial symbionts, (*iii*) red genotypes harbouring *Rickettsiella* and becoming green at adulthood and (*iv*) red genotypes co-infected with *Rickettsiella* and *Hamiltonella* and becoming green at adulthood. For each aphid type, two or three different aphid genotypes were considered. These strains were reared on *Vicia faba* plants as monoclonal populations. Experimental aphids were used between 24 hours and 48 hours after reaching adulthood to guarantee both no reproduction during the experiment and the symbiont-mediated colour change in *Rickettsiella*-infected strains. To synchronize individuals for the experiments, about five to ten adult aphids were isolated from mass rearing and able to reproduce for one day. Their offspring were isolated, maintained, monitored daily and used for experiments.

**Table 1:** Aphid strains used in the study. Letters reported in the ‘Symbiotic status’ column stand for the symbiotic complement including the obligate symbiont *Buchnera* (B) and the two facultative symbionts, *Rickettsiella* (R) and *Hamiltonella* (H).

Aphid type	Aphid genotype name	Origin	Reference	Colour		Symbiotic status	Strain code
				Larva	Adult		
Aphids with no facultative symbionts and being naturally red (R_B_)	JML06	Jena (D) 2006	[40]	Red	Red	B	R_B_—1
	LSR1	New York (USA) 2007	[41]	Red	Red	B	R_B_—2
	L3Lc_03	Bugey (FR) 2011	[42]	Red	Red	B	R_B_—3
Aphids with no facultative symbionts and being naturally green (G_B_)	Colmar	Colmar (FR) 1972	[43]	Green	Green	B	G_B_—1
	P123	Rennes (FR) 1999	[43]	Green	Green	B	G_B_—2
	LL01	Lusignan (FR) 1989	[43]	Green	Green	B	G_B_—3
Aphids infected with *Rickettsiella* and becoming green at adulthood (G_BR_)	L9Ms_18	Bugey (FR) 2011	[42]	Red	Green	BR	G_BR_—1
	L14Os_06	Bugey (FR) 2011	[42]	Red	Green	BR	G_BR_—2
Aphids coinfected with *Rickettsiella* and *Hamiltonella* and becoming green at adulthood (G_BRH_)	RA04	Rennes (FR)	[17]	Red	Green	BHR	G_BRH_—1
	L13Ma_03	Bugey (FR) 2011	[42]	Red	Green	BHR	G_BRH_—2

#### Ladybirds

*Coccinella septempunctata* ladybirds were provided by a grower (Créa) and maintained on a mixture of pea aphid genotypes that differ from those tested in the experiments. Pupae were isolated from mass rearing and emergence was daily checked. One to three days from emergence as adult, ladybirds were starved for 24 hours before the experiment as starvation enhances prey searching activity [22]. This experimental procedure allowed us to standardize the status of predator individuals and to maximize foraging activity [23].

All insects were maintained under a long day regime (16 h of light) in climate rooms (20°C, 70%±10% relative humidity).

### Predation assays

**Fig 1:**
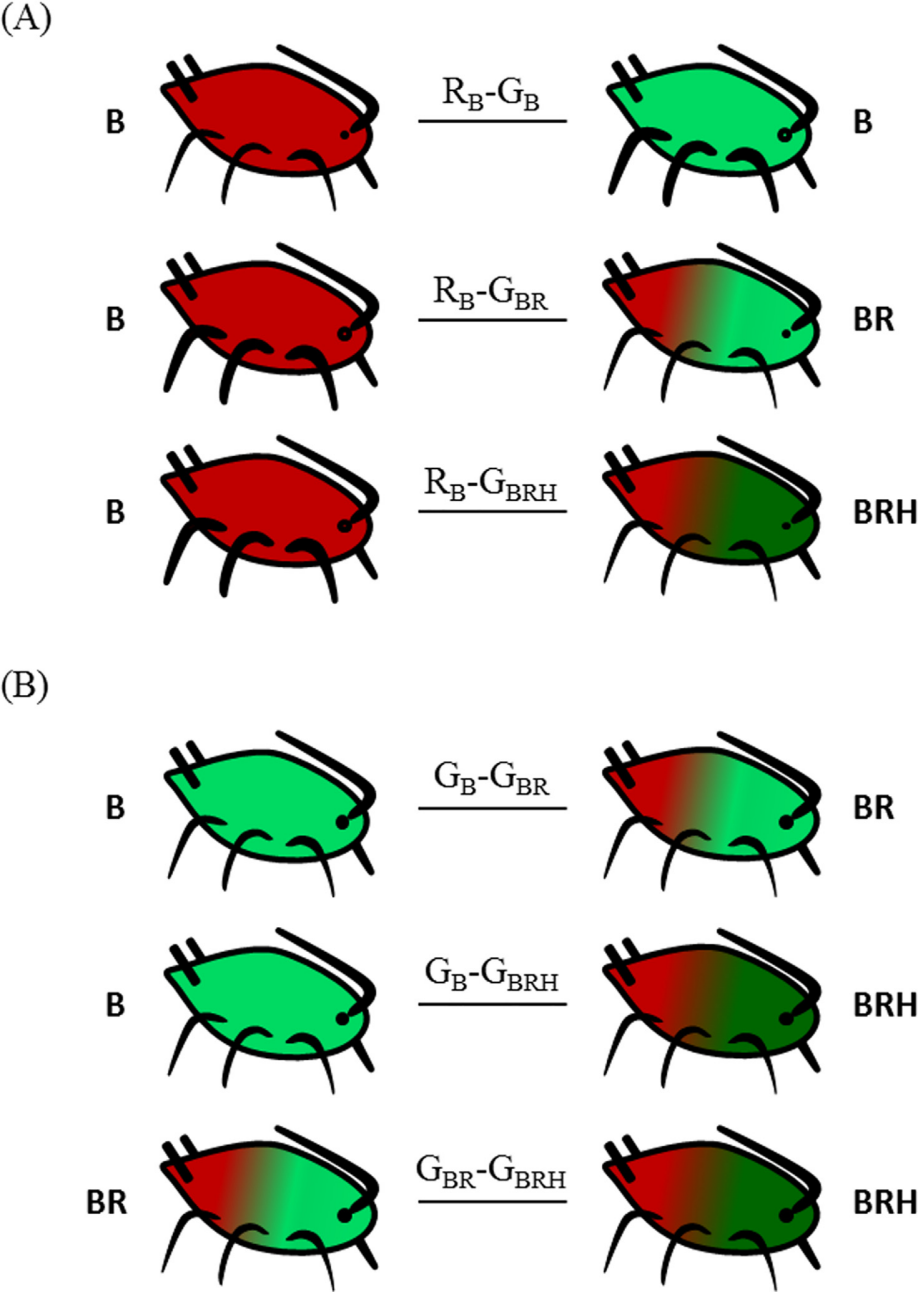
The experimental treatments. The six experimental treatments combining pairs of aphid types in order to test the effects of colour and symbiotic complement on aphid survival under predation pressure. Aphid type was defined as a combination of aphid colour and symbiotype. The aphid survival rate was tested (A) between red and green aphid types with different symbiotic complement and (B) among green types differing by their symbiotic complements. Letters reported in the aphids stand for the symbiotic complement including the obligate symbiont *Buchnera* (B) and the two facultative symbionts, *Rickettsiella* (R) and *Hamiltonella* (H). The name code of each treatment is indicated on the link between considered aphid types (Capital letter: the aphid colour; Subscript letters: symbiotic complement).

Each experiment consisted of a potted *Vicia faba* plant containing thirty aphid individuals: fifteen individuals from one aphid type and fifteen individuals from another aphid type. For each replicate, the genotype used for a given aphid type was randomly chosen among the two or three available ones (see Table 1). The thirty aphid individuals were placed on the plant one hour before the experiment for their settlement. A ladybird was then introduced in the experimental system and the plant was covered with a punctured plastic bag in order to avoid insect escape and external disturbance. Twenty-four hours later, the predator was extracted from the trial and the surviving aphid individuals (i.e., not consumed by the predator) of the two aphid types were counted. When the experiment considered only green aphids with different symbiotic status, their distinction was allowed by symbiotyping the surviving individuals (diagnostic PCR). For more details on extraction, PCR procedure or electrophoresis of PCR products, see [17]. Fig 1 presents the six aphid type combinations (i.e., treatments) tested. Three treatments combined red and green adult aphids with different symbiotic complements and three other treatments combined green adult aphids with various symbiotic consortia. For each treatment, twenty replicates were done (except for treatment G_BR_-G_BRH_ where the number of replicates was 16).

All experiments were done under a long day regime (16 h of light) in climate rooms (20°C, 70%±10% relative humidity).

### Statistical analysis

Two statistical analyses were conducted. First, we analysed whether the overall rate of survival, defined as the number of surviving aphids among the thirty previously installed, varied among the six experimental treatments. For this statistical analysis, the overall survival rate was tested against the experimental treatment, defined as a fixed factor and the aphid genotypes used in the experiment, defined as a random factor. As all aphids of a given experiment were exposed to the same predator individual, the ladybird individual was treated as a random factor in our statistical modelling. In case of significance of the experimental treatment, we performed pairwise comparisons between the six levels of this factor. Secondly, we analysed whether ladybird exhibited a preference when exposed to two aphid types. The dependent variable was the survival rate defined as the number of surviving aphids among the fifteen for each aphid type. For each experimental treatment, the aphid survival rate was tested against the aphid type, defined as a fixed effect, and their genotype, defined as a random factor. The ladybird was also included in the statistical modelling as a random factor. For both statistical analyses, we fitted a generalized linear mixed model (GLMM) assuming a Binomial error and a logit-link function. These GLMMs were fitted using the *lme4* package [24] in R 3.1.1 [25]. To assess the significance of the fixed model term, we used a likelihood ratio test. The pairwise contrasts were performed with the function *esticon* in the *doBy* package. Given the objectives of our study, only the effect of the fixed factor (i.e., experimental treatment (first analysis) and the aphid type (second analysis)) on the dependent variables will be detailed.

## Results

**Fig 2:**
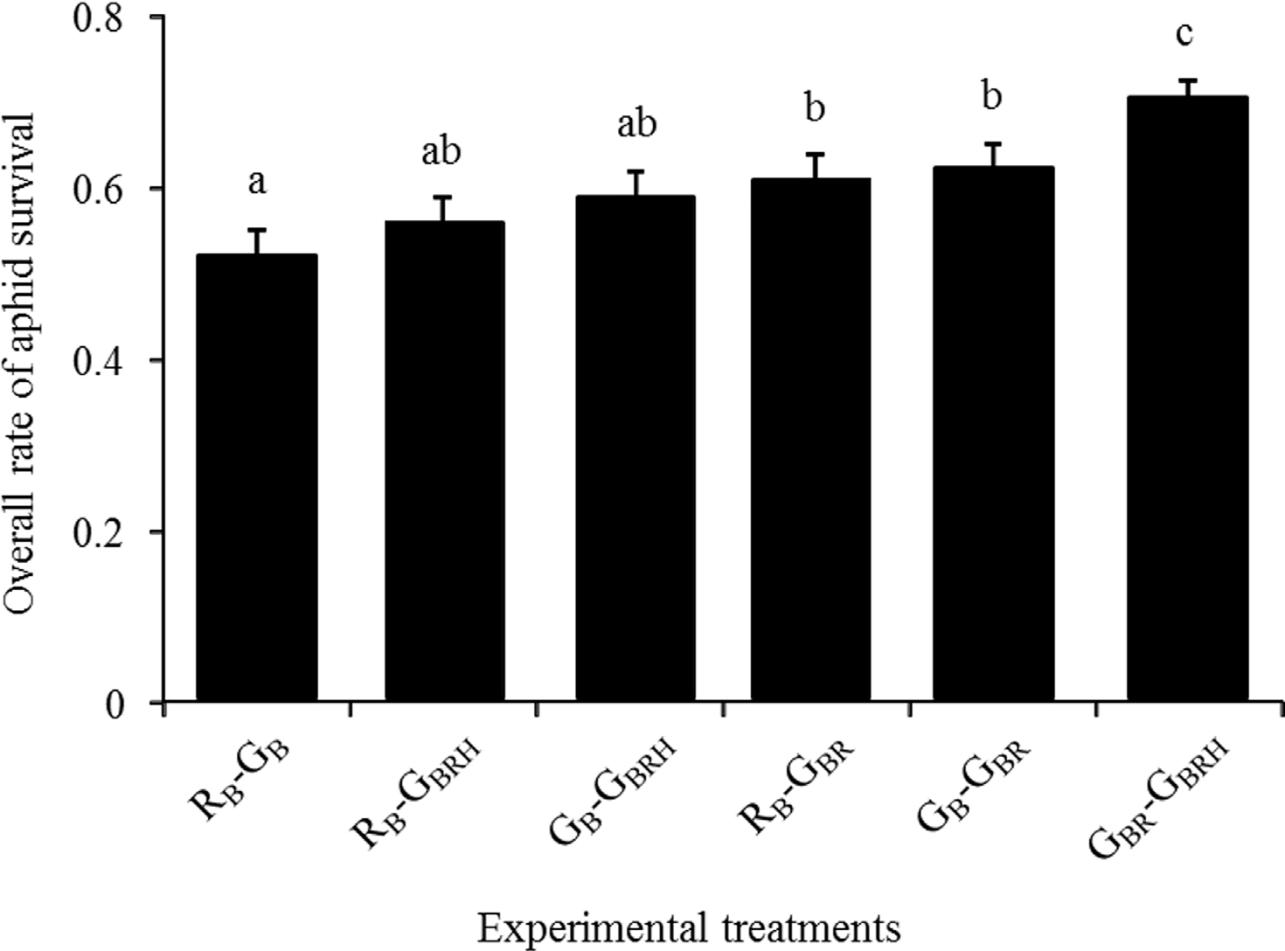
The overall rate of aphid survival. Proportion of surviving *Acyrthosiphon pisum* aphids among the thirty exposed to predation by an adult *Coccinella septempunctata* during 24 hours. Each treatment is the combination of two aphid types exposed to predation. See Fig 1 for the treatment code. Twenty replicates have been conducted per treatment (except for treatment G_BR_-G_BRH_ where N = 16). Error bars represent the standard error of the proportion. Different letters presents significant difference (p < 0.05; GLMM).

According to the first analysis, the overall rate of aphid survival when exposed to ladybird predation varied among the six experimental treatments (χ^2^ = 15.89; df = 5; p = 0.007; Fig 2). The level of ladybird consumption was lowest when we combined aphid individuals infected with *Rickettsiella* and aphid individuals co-infected with both facultative symbionts (overall rate of aphid survival: 0.70±0.04). Inversely, the rate of predation was significantly highest when predators were exposed to aphids free of facultative symbionts (overall rate of aphid survival: 0.51±0.03). Globally, the overall rate of aphid survival was highest in the three experimental treatments including individuals harbouring *Rickettsiella* singly (Fig 2) whatever the colour of the aphids exposed to ladybird predators.

**Fig 3:**
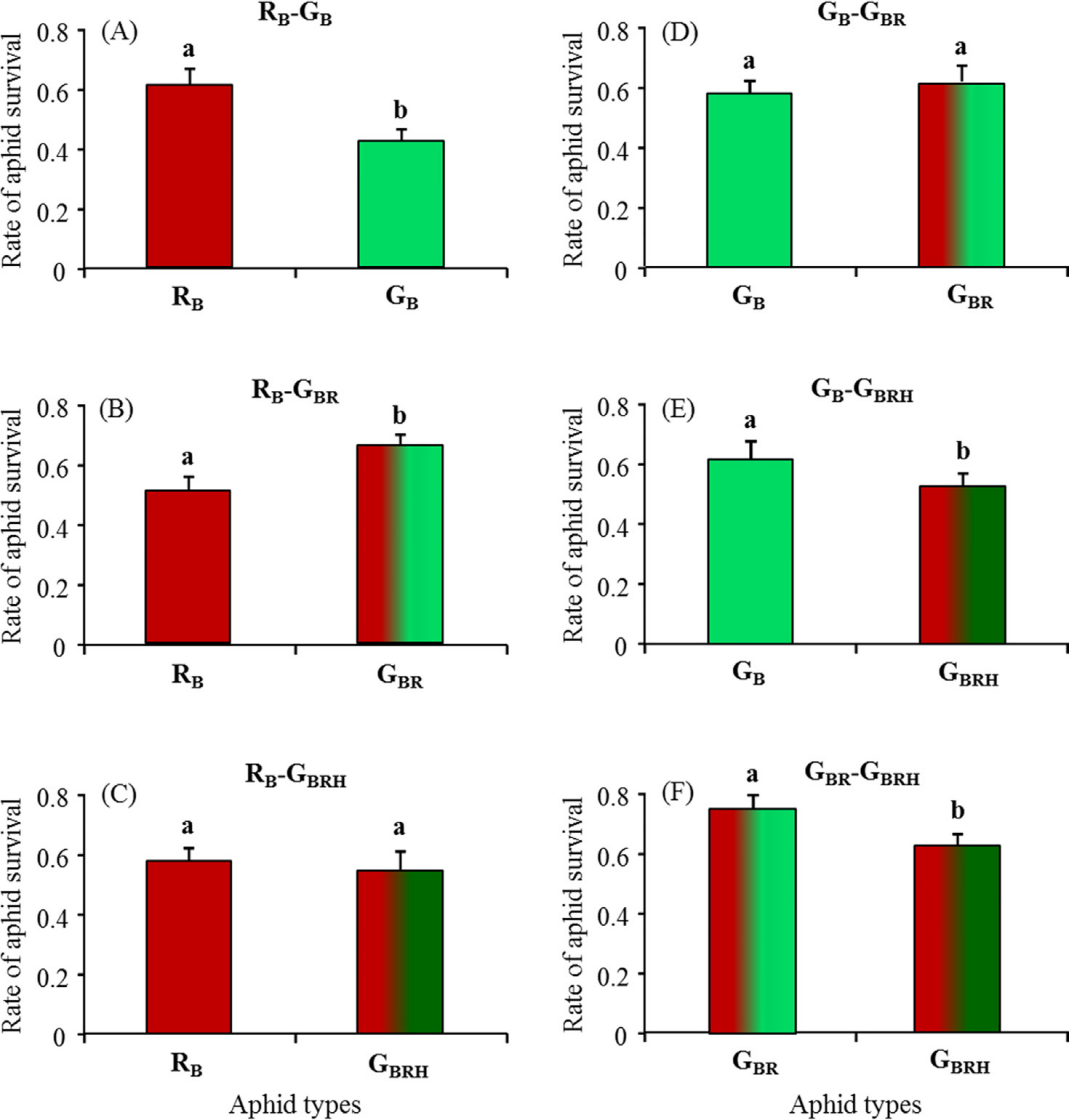
The survival rate of aphids depending on their type. Proportion of surviving *Acyrthosiphon pisum* aphids among fifteen of each type exposed to predation by an adult *Coccinella septempunctata* during 24 hours. Each treatment is the combination of two aphid types exposed to predation. Twenty replicates have been conducted per treatment (except for treatment G_BR_-G_BRH_ where N = 16). (A)–(F): results for each experiment treatment. Error bars represent the standard error of the proportion. Different letters presents significant difference (p < 0.05; GLMM).

When the ladybirds faced red and green adult aphids, the rate of predation on the red individuals depended on the symbiotic status of the green ones. When all aphids were free of facultative symbionts, ladybirds consumed significantly more green aphids than red ones (χ^2^ = 25.14; df = 1; p<0.001; Fig 3(A)) (survival rate of red aphids: 0.64±0.05; survival rate of green aphids: 0.43±0.04). Inversely, when ladybirds attacked a combination of red and *Rickettsiella*-infected green aphids, the latter were less consumed (χ^2^ = 17.27; df = 1; p<0.001; Fig 3(B)) (survival rate of red aphids: 0.52±0.05; survival rate of *Rickettsiella*-infected green aphids: 0.68±0.03). Finally, the aphid survival rates were similar between the red uninfected aphids and the green aphids co-infected with *Rickettsiella* and *Hamiltonella* (χ^2^ = 0.63; df = 1; p = 0.427; Fig 3(C)).

When ladybirds faced only green adult aphids, the survival rate of the individuals depended on their symbiotic complement. When combined, naturally uninfected green aphids and *Rickettsiella*-infected green aphids suffered from the same level of predation (χ^2^ = 0.94; df = 1; p = 0.331; Fig 3(D)). However, the green aphids co-infected with *Hamiltonella* and *Rickettsiella* had lower survival rates when combined with naturally green aphids (χ^2^ = 140.14; df = 1; p<0.001; Fig 3(E)) or *Rickettsiella*-infected green aphids (χ^2^ = 10.38; df = 1; p<0.001; Fig 3(F)).

## Discussion

Symbiotic associations may provide extended phenotypes to their hosts that could change their relationship with natural enemies [3]. In the pea aphid, the red individuals infected with *Rickettsiella* change their body colour becoming green at adulthood and our study showed that this symbiotic association influenced the survival rate of the host when exposed to predators. At the host population level, whatever the colour of aphid individuals within the population, the predator consumption rates significantly declined when the aphid population contained *Rickettsiella*-infected individuals (Fig 2). At the host individual level, red aphids were less consumed in presence of naturally green individuals whereas they suffered highest predation when they cohabit with *Rickettsiella*-infected green individuals (Fig 3). Our results thus suggest that the infection with *Rickettsiella* can be beneficial for pea aphids as it may confer a protection toward predation. However, this symbiont-mediated protection against predation would be conditional as the multiple infections with *Rickettsiella* and *Hamiltonella* decreased the *Rickettsiella* beneficial effect.

The effect of *Rickettsiella* infection on the survival rate of aphids exposed to predators may be explained by two non-exclusive mechanisms: the symbiotic complement of aphids would influence their detection by *C*. *septempunctata* ladybirds and/or their profitability for the predator.

### Symbiotic complement and prey detection

The most frequent sensory systems used to detect a prey include the olfactory and visual systems. The ladybird *C*. *septempunctata* has photoreceptors in the UV, blue, and green, suggesting that this coccinellid is sensitive to green stimuli and that it has the ability to distinguish between red and green colours [21]. Colour cues have a significant role on the foraging behaviour of *C*. *septempunctata*, whereby more red than green aphids are usually consumed [20], [21]. Surprisingly, in the experimental treatment containing red and green aphids free of facultative symbionts (i.e., treatment R_B_-G_B_), we showed that *C*. *septempunctata* attacked more green than red individuals. In [20] and [21], one genotype per colour morph was used and the authors did not control the symbiotic complement of aphids as at that time, only *Rickettsia* sp and *Serratia symbiotica* facultative symbionts were reported in pea aphid, without any idea about their effects on host phenotypes [26], [27]. Also, the predation exposure duration was short (30 minutes in [21] and 4 hours in [20]). Here, different red and green aphid genotypes, with controlled symbiotic complement, were exposed to ladybirds during 24 hours under a day/night regime as brightness impacts the predation rate [21]. The differences in experimental procedures would explain contrasting results between our and previous studies and the supported idea that red aphids are more exposed to predation must be reconsidered.

In line with [20] and [21], red aphids were more consumed in the present study but only in presence of *Rickettsiella*-infected green aphids. Naturally green aphids show a more greenish hue than *Rickettsiella-*infected ones (Tsuchida et al., unpublished data) and this difference in aphid colour may explain the variation of ladybird foraging activity. Nevertheless, once together, naturally green and *Rickettsiella*-infected aphids were consumed equally. Hence, the ladybirds would detect both aphid types similarly suggesting that the colour cues would not be the most relevant mechanism explaining the protection effect of *Rickettsiella* infection toward the ladybird predation.

In addition to visual cues, adult *C*. *septempunctata* can also use olfactory cues for aphid detection from a long distance [28], [29]. The alarm pheromone, i.e., E-*β*-farnesene (EBF) emitted by aphids is an effective kairomone for *C*. *septempunctata* [30]. Furthermore, the aphid symbiotic complement is known to impact the amount of EBF produced (lower EBF level in whole body extracts from *Hamiltonella* aphids) [31]. From our experimental set-up, no olfactory cues can be inferred. Further works are needed to analyse semiochemicals produced by aphids with different symbiotic complement in order to find volatiles affecting negatively the ladybird searching behaviour and acting as a ‘chemical shield’.

### Symbiotic complement and prey profitability

Once detected, a prey can be more or less profitable. First, the profitability of a prey item depends on ecological variables such as the time and energy required for the prey capture. Once attacked by an enemy, pea aphids present prevalent defensive behaviours like aggressiveness towards the enemy or escape reactions [32]. These behaviours that deter the enemies can be affected by the symbiotic status of the aphids: individuals harbouring *Hamiltonella* or co-infected with *Hamiltonella* and PAXS (i.e., Pea Aphids X-type Symbiont) expressed less defensive behaviours and suffer higher parasitism and predation rates than symbiont-free congeners [33], [34]. As facultative symbionts of the pea aphid can modulate host’s behaviours, the protective effect of *Rickettsiella* infection toward *C*. *septempunctata* adults could be associated with a *Rickettsiella*-induced increase in defensive behaviours. Future work should assess if *A*. *pisum* individuals infected with *Rickettsiella* exhibit more escape reactions or/and highest aggressiveness towards the enemy.

Second, the profitability of a prey depends on its palatability and toxicity. In some systems, symbionts negatively impact predators as the microbes reduce predation through the production of either predation-deterring toxins [8] or growth inhibiting molecules [35]. Symbionts can also affect indirectly the predation by reducing the survival of the predator after predation. In the pea aphid, the two facultative symbionts *Serratia* and *Hamiltonella* seem to reduce the aphid nutritional quality for the ladybird *Hippodamia convergens*: the predatory larvae fed on aphids with facultative symbionts had reduced survival [36].

From these recent results, a reliable explanation of the observed decline of predator consumption rates when the pea aphid population contained *Rickettsiella*-infected individuals (Fig 2) is that *Rickettsiella* infection would decrease the quality of the aphids (i.e., behavioural defence and/or unpalatability/toxicity) and by modifying the predator’s profitability, the *Rickettsiella* symbiotic association would deter ladybird feeding.

### Multiple infections and host fitness

*Rickettsiella* may benefit their hosts ecologically but this beneficial effect would be conditional as it depends on the presence of other facultative symbionts in hosts. In nature *Rickettsiella* is commonly found in association with *Hamiltonella* [16]. However the co-infection is responsible for high fitness costs on the aphid host in terms of longevity and reproduction [17]. Here, aphids hosting *Hamiltonella* and *Rickettsiella* were more consumed by ladybirds compared to aphid infected with *Rickettsiella* singly. As said before, individuals harbouring *Hamiltonella* singly or co-infected with *Hamiltonella* and PAXS expressed less defensive behaviours and are more exposed to parasitism and predation [33], [34]. The co-occurrence of *Hamiltonella* and *Rickettsiella* would also have an underlying ecological cost. Moreover, the density of *Rickettsiella* within aphid individuals declines in case of multiple infections with *Hamiltonella* (Polin et al., unpublished data) and this density reduction could in turn decrease the beneficial effect conferred by *Rickettsiella*. From these different results, the maintenance of this co-infection status in host populations could not be understood as it appears mostly costly for the host. One could hypothesize that this symbiotic status is only transitory after association during sexual reproduction [37] or horizontal transmission [38]. Or, the maintenance of this *Hamiltonella*-*Rickettsiella* association might be explained by other ecological conditions and far-reaching effects of symbionts unexplored until now.

The pea aphid symbiont system may be manipulated in such a way that the effect of symbiont infections may be compared in a common host genetic background. In the present study, the pea aphid strains used were collected from the field and these aphids in their naturally infected combinations have different genotypes. This experimental design implies that the present study is more correlative than causative. The protective effect of the bacterial symbiont *Rickettsiella* in pea aphids has thus to be confirmed by using aphid lineages with manipulated symbiotic infection status.

### Conclusion

Until now, the only reports of predation impacted by prey body colour change were the parasitic manipulations of crustacean hosts by acanthocephalan parasites enhancing transmission to their definitive host [39] and the bioluminescence in the bobtail squid by the bacterium *Vibrio fischeri* producing a camouflage from predators [9]. In the pea aphid, the infection with the bacterial symbiont *Rickettsiella* impacts positively the host survival by reducing predation risk under specific conditions. This beneficial effect would be rather associated to symbiotic change in aphid profitability than the symbiotic-mediated colour change. As the predatory ladybirds are deterred from eating aphids in presence of aphids singly infected with *Rickettsiella*, the predation risk is reduced for the nearby conspecifics. This predation symbiotic protection would not have associated fitness costs as aphid longevity and reproduction are not affected by *Rickettsiella* infection [17]. In the case of co-infection the benefits of this extended phenotype is however totally hidden by the cost of additionally harbouring *Hamiltonella*. The maintenance of the *Rickettsiella* symbiosis is then modulated by the host ecological interactions (i.e. predation) and the symbiont ecological interactions (i.e. co-infection). This study demonstrates the necessity to consider the ecological network as a whole to understand each ecological interaction further. Finally, further studies are necessary for identifying the mechanisms of the *Rickettsiella*-mediated protection and its specificity towards the other aphid predators (e.g. lacewings, hoverflies). Overall, such studies will precise the potential incidences of microbial symbioses on the food webs properties in the ecosystems. Due to the use of ‘natural’ aphid strains, this study is more correlative than cause and effect. The variability between symbiont effects, in particular the significant variations among pea aphid clones infected with different bacterial complement, deserves further attention.
